# Draft Genome Sequence of Methicillin-Resistant *Staphylococcus aureus* Strain SA16, Representative of an Endemic Clone from a Brazilian Hospital

**DOI:** 10.1128/genomeA.00754-13

**Published:** 2013-09-19

**Authors:** Andrei Nicoli G. Dabul, Veronica N. Kos, Michael S. Gilmore, Ilana L. B. C. Camargo

**Affiliations:** Instituto de Física de São Carlos, Universidade de São Paulo, São Carlos, São Paulo, Brazila; Departments of Ophthalmology and of Microbiology and Immunobiology, Harvard Microbial Sciences Initiative, Harvard Medical School, Massachusetts Eye and Ear Infirmary, Boston, Massachusetts, USAb; The Broad Institute, Cambridge, Massachusetts, USAc

## Abstract

Here we report the draft genome sequence of a bloodstream isolate of methicillin-resistant *Staphylococcus aureus* strain SA16. Strain SA16 is a sequence type 5 (ST5)-staphylococcal cassette chromosome *mec* type II (SCC*mec* II) clone and was the most prevalent isolate at a Brazilian hospital during the second half of 2009.

## GENOME ANNOUNCEMENT

Methicillin-resistant *Staphylococcus aureus* (MRSA) is an important nosocomial pathogen for which tracking is required to detect outbreaks.

An epidemiological study of MRSA isolated from infection sites in patients at a hospital in Belo Horizonte, Brazil, between July and December 2009 revealed the presence of an endemic sequence type 5 (ST5)-staphylococcal cassette chromosome *mec* type II (SCC*mec* II) clone (1) that is different from the ST239-SCC*mec* III strains typically encountered in Brazilian hospitals (2). The aim of this study was to characterize by whole-genome sequencing a MRSA isolate representative of this circulating clone. Strain SA16 was isolated from a patient’s blood and identified as MRSA by use of the Vitek 2 (bioMérieux) system. Use of the Kirby-Bauer protocol showed this strain to be susceptible to vancomycin, amikacin, and gentamicin but resistant to oxacillin, ciprofloxacin, clindamycin, erythromycin, and penicillin. According to an Etest (bioMérieux), SA16 was also susceptible to daptomycin (MIC, 1 mg/liter), teicoplanin (MIC, 3 mg/liter), linezolid (MIC, 0.75 mg/liter), and tigecycline (MIC, 0.19 mg/liter) and had intermediate susceptibility to quinupristin-dalfopristin (MIC, 1.5 mg/liter).

Genomic DNA was extracted and purified and then submitted for Illumina HiSeq (Illumina) next-generation sequencing at the Tufts University DNA Core Facility (Boston, MA). Independently, genomic DNA was also subjected to 454 FLX (Roche) analysis at the University at Buffalo Next-Generation Sequencing and Expression Analysis Core (Buffalo, NY) to obtain coverage greater than 10-fold. The reads produced by both platforms were assembled into contigs by mapping them against a reference genome sequence (*S. aureus* N315) using CLC Genomics Workbench v 4.8.

Unmapped reads were then assembled into contigs by *de novo* assembly. The contigs of >200 bp (*n* = 74) were annotated with the Prokaryotic Genome Annotation Pipeline v2.0 (NCBI) for deposition with GenBank. The draft genome sequence consists of 2,961,555 bp, with a GC content of 32.9%. Compared to the reference N315 genome sequence, 99% of the genome sequence was found to be shared.

Sequence analysis allowed classification of the clone as *agr* type II, *cap* type 5, and *spa* type t539. SA16 was found to harbor a typical virulence factor associated with CC5 strains (3–18). SA16 was also found to harbor *msr*A, *msr*B, *erm*A, and *erm*C genes, all related to streptogramin type B resistance (19). This may explain the intermediate level of resistance to quinupristin-dalfopristin. *erm*C is probably located on a plasmid, since it is present on a contig which does not match with the *S. aureus* N315 genome but aligns to various plasmids. SA16 also contains 2 intact phages, φMR25 and φN315. φN315 was also found in the N315 genome.

Sequencing of the *S. aureus* SA16 genome has generated the first draft genome sequence of a representative CC5 strain from Brazil, which provides a reference for further comparative genome analysis for studies of *S. aureus* isolates collected within this region. Also, this strain has been used by our group in the study of novel antimicrobial compounds and the structural study of *S. aureus* proteins.

### Nucleotide sequence accession numbers.

This whole-genome shotgun project has been deposited at DDBJ/EMBL/GenBank under the accession number ASZO00000000. The version described in this paper is version ASZO01000000.
